# Assessment of the duration and effectiveness of intra-articular lidocaine injections for groin pain in patients with labral tears involving early osteoarthritis

**DOI:** 10.1051/sicotj/2020049

**Published:** 2021-01-12

**Authors:** Kensuke Fukushima, Gen Inoue, Ayumu Kawakubo, Kentaro Uchida, Tomohisa Koyama, Yoshihisa Ohashi, Katsufumi Uchiyama, Naonobu Takahira, Masashi Takaso

**Affiliations:** 1 Department of Orthopaedic Surgery, Kitasato University School of Medicine 1-15-1 Kitasato Minami-ku Sagamihara Kanagawa 252-0374 Japan; 2 Department of Rehabilitation, Kitasato University School of Allied Health Sciences 1-15-1 Kitasato Minami-ku Sagamihara Kanagawa 252-0373 Japan

**Keywords:** Labral tear, Intra-articular injection, Hip arthroscopy, Lidocaine

## Abstract

*Introduction*: Intra-articular lidocaine injections have been used to confirm the hip pathology and may predict the efficacy of arthroscopic surgery. We have routinely performed the injections as a surgical indicator. The aim of this study was to assess the duration and effectiveness of these diagnostic intra-articular lidocaine injections on groin pain in patients with labral tears involving early osteoarthritis. *Methods*: A total of 113 patients were included in this study. All patients received one injection of 10 ml of 1% lidocaine into the hip joint under fluoroscopy. The duration and effectiveness of the injection were assessed 2 weeks after the injection and at a minimum of 1 year of follow-up. The effect of the injection was graded as 0: unchanged or worse; 1: an effect only on the day of injection; 2: the effect lasted a few days; 3: the effect lasted about a week; and 4: symptom remission. In addition, we recorded whether hip arthroscopic surgery was eventually performed. *Results*: The effect was rated as 0 in 19 patients (16.8%), as 1 in 30 patients (26.5%), as 2 in 38 patients (33.6%), as 3 in 13 patients (11.5%), and as 4 in 13 patients (11.5%). Seventy-two patients (63.7%) underwent hip arthroscopic surgery. No relationship with patients’ characteristics was found. *Conclusion*: In total, 83% of patients experienced some effect of the lidocaine injection. Furthermore, 11.5% of patients experienced complete remission of their symptoms.

## Introduction

In recent years, hip arthroscopy has become a common surgical procedure in both the diagnosis and treatment of painful hip disorders. Arthroscopic hip treatments typically result in good clinical outcomes, with low rates of complications and relatively rapid rehabilitation [1, 2]. However, pain around the hip joint may originate from different locations, including extra-articular origins and the lumbar spine [3–5]. Generally, arthroscopic treatments should be targeted at patients with pain of an intra-articular source. Thus, intra-articular lidocaine injections might be useful as a method to confirm the hip pathology and predict the efficacy of hip arthroscopic surgery. Consistent with this, several reports have described the usefulness of preoperative diagnostic intra-articular injections [6–8].

We have routinely performed diagnostic intra-articular lidocaine injections in patients who are considered candidates for hip arthroscopic surgery. Based on our experience, the duration of the effect of the injection differed among patients. Additionally, some patients feel unaffected by the injection despite showing obvious intra-articular abnormal findings. However, there are no reports with an assessment of the duration and effectiveness of intra-articular injection using a consistent protocol, with fixed medication and dose. Thus, the aim of this study was to investigate the duration and effectiveness of diagnostic intra-articular lidocaine injections on groin pain in patients with labral tears involving early osteoarthritis (OA).

## Patients and methods

We obtained approval from our Institutional Review Board (IRB) and the study was performed in accordance with the ethical standards laid down in the 1964 Declaration of Helsinki and its later amendments.

In this retrospective study, we included 113 patients (113 hip joints, male 46, female 67, mean age 44.3 ± 1.6 years old) who met the following criteria: diagnosed with a labral tear without acetabular dysplasia (i.e., lateral centre-edge (LCE) angle < 20°) by plain radiograph and magnetic resonance imaging (MRI); underwent intra-articular lidocaine injections for this condition between 2012 and 2016, and had a minimum of 1 year of follow-up after the injection. Patients with OA classified as Tönnis grade 3, septic arthritis, inflammatory diseases such as rheumatoid arthritis or purulent arthritis, or synovial chondromatosis were excluded. All patients complained of groin pain and restricted hip motion in flexion and internal rotation and had a positive anterior impingement test. None of the patients took analgesics, non-steroidal anti-inflammatory drugs (NSAIDs), or glucocorticoids during the examinations (i.e., 1 month before injection and 1 month after injection). The mean follow-up duration was 52.6 months.

For all patients, data regarding age, sex, anteroposterior (AP) and cross-lateral plain radiographic findings, and MRI findings at the time of the injection were collected. We evaluated the records for the extent of OA using the Tönnis classification [9] and radiological findings related to femoroacetabular impingement (FAI). The latter included the presence of coxa profunda, protrusio acetabuli, and an LCE angle ≥ 40°, which are indicators of a deep socket, and the crossover sign on the AP radiograph, which indicates acetabular retroversion. In addition, the femoral alpha angle was measured on a cross-lateral plain radiograph, and hip joints with a femoral alpha angle ≥ 55° were classified as having cam deformity. Hip joints with a deep socket, acetabular retroversion, and/or cam deformity were diagnosed as FAI [10].

After establishing the clinical diagnosis, based on physical and radiographical findings (i.e., plain radiographs and MRI), as a labral tear with or without early OA, all patients underwent one injection of 10 ml of 1% lidocaine into the hip joint under fluoroscopic guidance. Two weeks after the injection, all patients were evaluated for the duration and effect of the injection on their groin pain at our outpatient clinic. The duration and effectiveness were graded as follows: 0: unchanged or worse, 1: an effect only on the day of injection, 2: the effect lasted a few days, 3: the effect lasted about 1 week, and 4: symptom remission (Table 1). Complications related to the injection were also reviewed.

**Table 1:** Grading criteria for the evaluation of the duration and effectiveness of the intra-articular lidocaine injection on groin pain.

0	Unchanged or worse
1	Effect only on the day of injection
2	Effect lasted a few days
3	Effect lasted about 1 week
4	Symptom remission

### Statistical analysis

Statistical analyses were performed using JMP statistical software version 11.0 (SAS Institute Inc., Cary, NC, USA). Since age, sex, and incidence of radiographic findings of FAI might affect OA grading, we assessed the relationships between these parameters, as well as potential relationships between the effect of the injection and these parameters. Additionally, we evaluated the relationship between the effectiveness of the injection and subsequent hip arthroscopic surgery (during the follow-up period). For normally distributed data (i.e., age), the nonparametric Mann-Whitney *U* test was used for comparisons between groups. Sex, the incidence of radiographical findings of FAI, OA grading, and incidence of hip surgery were statistically compared between groups using the χ^2^ test. *P* values < 0.05 were considered as statistically significant.

## Results

In the radiological investigation, radiological findings related to FAI were observed in 41 (36.2%) patients. The pincer deformity was observed in 9 patients, cam deformity was observed in 8 patients, and both deformities were observed in 24 patients. Radiographic OA was graded as 0 in 73 hips (64.6%), as 1 in 38 hips (33.6%), and as 2 in 2 hips (1.8%). As very few patients had radiographic OA classified as grade 2, we evaluated the differences in patient characteristics (i.e., age, sex, and radiographic findings of FAI) between patients with early OA (i.e., grades 1 and 2) and those with grade 0; the results are shown in Table 2. Although age was significantly higher in patients with OA grade 1 and 2 than in patients with OA grade 0 (56.6 ± 1 2.9 vs. 37.6 ± 14.6, *P* < 0.001), there were no significant differences in sex (*P* = 0.0589) and incidence of radiographical findings of FAI (*P* = 0.842), including each type of deformity. There were no complications related to the intra-articular lidocaine injection in any of the patients.

**Table 2:** Relationship between osteoarthritis (OA) grade and the patients’ characteristics (*n* = 113).

OA grade	0 (*n* = 73)	1 and 2 (*n* = 40)	*P*-value
Age, mean ± SD (years)	37.6 ± 14.6	56.6 ± 12.9	<0.001*
Male (*n*)	25	21	0.0589
Female (*n*)	48	19	
Radiographic FAI (n (%))	26/73 (35.6%)	15/40 (37.5%)	0.842
Pincer type	5	4	
Cam type	6	2	
Mixed type	15	9	

* The Mann-Whitney *U* test or χ^2^ test showed a significant difference between the groups (*P* < 0.05).

The efficacy of the injection was rated as grade 0 in 19 patients (16.8%), as grade 1 in 30 patients (26.5%), as grade 2 in 38 patients (33.6%), as grade 3 in 13 patients (11.5%), and as grade 4 in 13 patients (11.5%) (Table 3). Overall, 83.2% of the patients experienced some effect of the injection. We further reviewed the patients who rated the effect as 4 (symptom remission); none of these patients experienced a recurrence of symptoms during a minimum of 1 year of follow-up.

**Table 3:** The number of patients according to the efficacy grading.

Efficacy grade	Patients (*n* (%))
0	19 (16.8%)
1	30 (26.5%)
2	38 (33.6%)
3	13 (11.5%)
4	13 (11.5%)

Table 4 indicates the relationships between the efficacy of the intra-articular lidocaine injection and the patients’ characteristics. In total, 72 patients (63.7%) subsequently underwent hip arthroscopic surgery during the follow-up period: seven patients (7/19: 36.8%) with an effect of grade 0; 26 patients (26/30: 86.7%) with an effect of grade 1; 33 patients (33/38: 86.8%) with an effect of grade 2; 6 patients (6/13: 46.2%) with an effect of grade 3; and no patients had an effect of grade 4. There was a statistically significant relationship between the effectiveness of the injection and the proportion of subsequent surgical application (*P* < 0.0001). The 7 patients with an effect of grade 0 showed an effect with a secondary intra-articular injection after pharmacological treatment with Pregabalin; however, their symptoms were not resolved and we subsequently performed hip arthroscopic surgery. In contrast, there were no significant relationships between the effectiveness of the injection and age, sex, radiographic findings of FAI, and the OA grade (*P* = 0.76, 079, 0.49, 0.10, respectively) (Table 4).

**Table 4:** Relationship between the efficacy of the intra-articular lidocaine injection and the patients’ characteristics (*n* = 113).

Efficacy grade	0	1	2	3	4	*P*-value
Arthroscopic surgery (*n* (%))	7/19 (36.8%)	26/30 (86.7%)	33/38 (86.8%)	6/13 (46.2%)	0/13 (0%)	<0.0001*
Age, mean ± SD (years)	49.1 ± 3.9	42.8 ± 3.1	43.4 ± 2.7	43.7 ± 4.7	44.2 ± 4.7	0.76
Male (*n*)	9	12	17	4	4	0.79
Female (*n*)	10	18	21	9	9	
Radiographic FAI (*n* (%))	7/19 (36.8%)	12/30 (40.0%)	16/38 (42.1%)	4/13 (30.8%)	2/13 (15.4%)	0.49
OA grade (*n*)						
0	14	21	23	5	10	0.10
1 and 2	5	9	15	8	13	

FAI: femoroacetabular impingement; OA: Osteoarthritis.

* The Mann-Whitney *U* test or χ^2^ test showed a significant difference among the groups (*P* < 0.05).

## Discussion

In the current study, we evaluated the duration and effectiveness of diagnostic intra-articular lidocaine injections on groin pain in patients with labral tears of the hip. In 83.2% of these patients, the intra-articular injection was effective to some degree. Additionally, the majority of patients who noted an effect of the injection subsequently underwent hip arthroscopic surgery. Since we performed the injection to confirm the accuracy of our clinical diagnosis and the appropriateness of the surgery, these results seem reasonable. Byrd and Jones demonstrated that the response to an intra-articular injection was 90% accurate in diagnosing an intra-articular hip abnormality [6]. Furthermore, a systematic review by Lynch et al. reported that the rate of pain relief after an intra-articular hip injection of a local anaesthetic among patients who complained of groin pain was 92.5% [11]. These efficacy rates are comparable to the rate in the present study, which further confirms the usefulness of intra-articular injections for confirming a diagnosis based on physical and radiological assessments, and as an indicator for hip arthroscopic surgery.

A previous systematic review reported a mean duration of pain relief of 2.35 days among patients with groin pain, but the detailed study designs of the included manuscripts were not consistent, and the effect size should vary according to the contents of the injection and its dose [11]. As far as we know, the present study is the first to assess both the duration and the effect size of intra-articular lidocaine injections using the same fixed-dose. In the present study, 55.6% of patients had an effect that lasted for more than 24 h. Moreover, the symptoms in 11.5% of patients were in remission after a single diagnostic injection. The average duration of the effect of 1% lidocaine when used for local anaesthesia or a caudal block has been reported as 24 and 89 min, respectively [12]. Although the effect of local anaesthesia in deep soft tissue tends to be prolonged, intra-articular lidocaine injections might have an effect that lasts beyond the duration of anaesthesia.

The mechanism underlying the long duration of the effect of intra-articular lidocaine, beyond the effect of local anaesthesia is still unclear. A previous study reported that patients affected by disc herniation might experience pain relief lasting a few weeks to months after the administration of lidocaine by a spinal nerve root block [13]. Ohtori et al. have shown, using an animal model of lumbar disc herniation, that lidocaine decreases acid-sensing ion channel (ASIC) 3 expression in dorsal root ganglion neurons for at least 12 days [14], suggesting that ASIC3 is related to the prolonged effectiveness of lidocaine. It has been reported that ASICs are the primary acid sensors in the nervous system, and among ASIC types, ASIC3 has been reported as being the most sensitive and predominantly distributed not only in the sensory peripheral nervous system, but also in nociceptors innervating the synovial joints, articular cartilage, growth plate, and meniscus, and in type B synoviocytes [15–19]. Because it has been reported that the expression of ASIC3 varies among individuals [17–19], the variations in the duration and effectiveness observed in the present study might be related to individual differences in the expression of ASIC3 in the hip joint. However, the mechanism is likely more complex, and further basic studies are warranted.

In the present study, we evaluated potential relationships between the efficacy of the intra-articular injection and age, sex, radiographic findings of FAI, and OA grade. One reason for the lack of a relationship between the OA and the effect of the injection might the inclusion of only patients with early OA in the present study. The systematic review by Lynch et al. reported that clinical and imaging findings were unreliable predictors of pain relief after intra-articular injections in patients who complained of groin pain [11]; this previous result is the same as that in the current study. Ayeni et al. assessed the relationship between the response to a preoperative intra-articular injection and the outcome after the arthroscopic management of FAI [20]. The authors concluded that, although a positive response was not a predictor of a positive outcome, a negative response might be a predictor of a negative outcome after hip arthroscopic surgery. Using a larger population than that in Ayeni et al., Krych et al. found that the response to the preoperative intra-articular injection might be a poor predictor of the surgical outcome [21]. Although we did not assess the clinical results after hip arthroscopic surgery, we similarly did not find any relationships between the patient characteristics and the efficacy of the intra-articular injection in the present study. We intend to re-examine these relationships in a larger population, with more detailed intra-articular findings from the hip arthroscopy, in the future.

Several limitations of the current study should be mentioned. First, this study comprised a single-arm, with no control group. In addition, the sample size was relatively small. Second, we did not assess the patients’ clinical condition using standard tools such as the visual analogue pain scale, Western Ontario and McMaster Universities Osteoarthritis Index (WOMAC), modified Harris hip score, and non-arthritic hip score (NAHS). Thus, prospective studies that use these tools and evaluate the outcomes of hip arthroscopic surgery, with large sample sizes, are needed in the future. Third, we did not use a contrast agent during the intra-articular injection because of allergy concerns. Therefore, there might have been cases that resulted in a false negative due to an extra-articular injection and/or leakage.

## Conclusion

In the present study, 83.1% of patients with labral tears reported some degree of efficacy of the intra-articular lidocaine injection. Furthermore, 55.6% of patients experienced an effect lasting more than 24 h, and 11.5% of patients achieved remission of their symptoms. Thus, intra-articular lidocaine injections might have potential beyond a diagnostic tool. We strongly recommend initially performing intra-articular lidocaine injections in patients with labral tears involving early OA who have groin pain and note the apparent radiographical findings on MRI.

## Funding

This research received no specific grant from any funding agency in the public, commercial, or not-for-profit sectors.

## Conflicts of interest

The authors declare that there is no conflict of interest.
